# Killer or helper? The mechanism underlying the role of adenylate activated kinase in sound conditioning

**DOI:** 10.3389/fnsyn.2022.940788

**Published:** 2022-09-07

**Authors:** Rui Zhao, Changhong Ma, Minjun Wang, Xinxin Li, Wei Liu, Lin Shi, Ning Yu

**Affiliations:** ^1^Department of Otorhinolaryngology, The First Affiliated Hospital of Dalian Medical University, Dalian, China; ^2^Department of Otolaryngology-Head and Neck Surgery, Ministry of Education, National Clinical Research Center for Otolaryngologic Diseases, The Sixth Medical Center of People’s Liberation Army (PLA) General Hospital, State Key Lab of Hearing Science, Beijing Key Lab of Hearing Impairment Prevention and Treatment, Beijing, China

**Keywords:** noise-induced hearing loss, sound conditioning, synapses, ATP-consumption, hair cell

## Abstract

**Objective:**

To investigate whether sound conditioning influences auditory system protection by activating adenylate activated kinase (AMPK), and if such adaption protects ribbon synapses from high-intensity noise exposure.

**Materials and methods:**

CBA mice (12 weeks old) were randomly divided into four groups (*n* = 24 mice per group): control, sound conditioning (SC), sound conditioning plus noise exposure (SC+NE), and noise exposure (NE). Hearing thresholds were assessed before testing, after sound conditioning, and 0, 3, 7, and 14 days after 110 dB noise exposure. Amplitudes and latencies of wave I at 90 dB intensity were assessed before test, after conditioning, and at 0 and 14 days after 110 dB noise exposure. One cochlea from each mouse was subjected to immunofluorescence staining to assess synapse numbers and AMPK activation, while the other cochlea was analyzed for phosphorylated adenylate activated kinase (p-AMPK) protein expression by western blot.

**Results:**

There was no significant difference in auditory brainstem response (ABR) threshold between SC and control mice. The degree of hearing loss of animals in the two SC groups was significantly reduced compared to the NE group after 110 dB noise exposure. Animals in the SC group showed faster recovery to normal thresholds, and 65 dB SPL sound conditioning had a stronger auditory protection effect. After sound conditioning, the amplitude of ABR I wave in the SC group was higher than that in the control group. Immediately after noise exposure (D0), the amplitudes of ABR I wave decreased significantly in all groups; the most significant decrease was in the NE group, with amplitude in 65SC+NE group significantly higher than that in the 85SC+NE group. Wave I latency in the SC group was significantly shorter than that in the control group. At D0, latency was prolonged in the NE group compared with the control group. In contrast, there was no significant difference in latency between the 65SC+NE and 85SC+NE groups. Further, at D14, there was no significant difference between the NE and control groups, while latency remained significantly shorter in the 65SC+NE and 85SC+NE groups compared with controls. Number of ribbon synapses in SC mice did not differ significantly from that in controls. After 110 dB noise exposure, there were significantly more ribbon synapses in the SC+NE group than the NE group. Ribbon synapses of all groups were recovered 14 days after the noise exposure, while the SC group had a shorter recovery time than the non-SC groups (*p* < 0.05). AMPK was highly activated in the SC group, and p-AMPK expression was detected; however, after 110 dB noise exposure, the strongest protein expression was detected in the NE group, followed by the SC+NE groups, and the lowest protein expression was detected in the control group.

**Conclusion:**

Sound conditioning animals were more noise resistant and recovered hearing faster than non-SC animals. Further, 65 dB SPL SC offered better hearing protection than 85 dB SPL SC. Early AMPK activation may protect hearing by increasing ATP storage and reducing the release of large quantities of p-AMPK, which could help to inhibit synapse damage.

## Introduction

Noise intensity and duration of exposure determine the level of noise damage to an organism. High-intensity noise exposure damages inner hair cells (IHCs), primarily through two pathways: direct mechanical damage, in which noise can destroy the static cilia of hair cells, resulting in hair cell loss and damage to supporting cells and spiral ganglia (Kapoor et al., 2019); and biochemical reactions, which cause hair cell apoptosis or necrosis (Kurabi et al., 2017). Oxidative stress, poor energy metabolism, inflammatory reactions, glutamate buildup, and calcium overload are all known to trigger noise-induced apoptosis in IHCs (Wu J. et al., 2020). Low-intensity noise can both cause hearing loss and strengthen noise resistance in an organism, where the latter is referred to as sound conditioning. Sound conditioning is defined as exposure to low-level, non-traumatic sound stimuli, prior to high-intensity noise exposure, which does not generate permanent threshold shift (PTS), but rather reduces the transient threshold shift (TTS) or PTS triggered by subsequent high-intensity noise (Suryadevara et al., 2009; Roy et al., 2013; Chen et al., 2014; Waqas et al., 2018). Hair cell protection, activation of the hypothalamic-pituitary axis (HPA) axis, oxidative stress, control of the auditory efferent neural system, and increase of cochlear microcirculation are among the mechanisms underlying the protective impacts of sound conditioning on hearing that have been proposed to date (Cody and Johnstone, 1982; Niu et al., 2003; Harris et al., 2006; Henderson et al., 2006; Tahera et al., 2007; Zuo et al., 2008; Maison et al., 2013; Alvarado et al., 2016); however, the entire repertoire of mechanisms involved in this process is not fully understood.

Adenylate activated kinase (AMPK) is a critical cellular energy sensor that regulates cellular and systemic energy homeostasis and may be involved in the relationship between neuronal activity and energy availability, as it responds rapidly to elevated intracellular AMP/ATP ratios, and controls food intake and peripheral energy expenditure in the hypothalamus (Xue and Kahn, 2006; Winder and Thomson, 2007). AMPK protects the central nervous system in patients with ischemic stroke by reducing oxidative stress, decreasing neuroinflammation, improving mitochondrial function, and suppressing glutamate excitotoxicity (Matthews and Fuchs, 2010; Liu et al., 2014; Qiu et al., 2016). Nevertheless, massive AMPK activation causes phosphorylation of AMP-activated protein kinase (p-AMPK), which activates c-Jun N-terminal protein kinase (JNK), causing apoptosis (Weisova et al., 2011). When CBA mice were exposed to white noise at 106 dB sound pressure level (SPL) for 2 h, a single massive activation of AMPK led to a significant hearing threshold shift and a reduction in IHC ribbon synapse release, while a 30% reduction in AMPK activation induced by application of antagonists reduced the degree of hearing loss by 80%, implying that excessive AMPK activation is also a relevant mechanism underlying noise-induced hearing loss (Hill et al., 2016). These findings suggest that AMPK performs a complex and multi-targeted role in noise-induced inner ear injuries. We hypothesized that AMPK activation acts as a “two-way switch,” in which activation of p-AMPK at a certain concentration causes death of inner ear cells, while below that threshold, AMPK is generally protective.

Noise levels between 55 and 100 dB SPL are proven to have protective effects, while it is difficult to produce sound conditioning effects with noise < 55 dB SPL, and levels > 100 dB SPL are likely to cause hearing loss; hence, noise intensities used in sound conditioning studies to date have generally been in the range 85–100 dB SPL (Canlon, 1997; Pukkila et al., 1997; Fan et al., 2020). In this study, the effects of sound conditioning on the auditory system were assessed by observing AMPK activation and ribbon synapse release during sound conditioning. Our findings provide a new theoretical basis for future strategies to prevent noise-induced deafness and cochlear synaptic disorder.

## Materials and methods

### Experimental animals and groups

**FIGURE 1:**
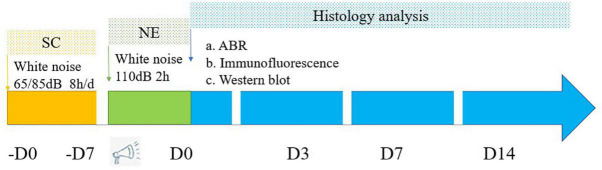
Flowchart depicts the major procedures of the experiment. SC, sound conditioning; NE, noise exposure; -D0 ∼ -D7, the 65SC and 85SC groups were exposed to white noise at 65 or 85 dB SPL, respectively, for 8 h per day for 7 days; D0, after sound conditioning and immediately post-acoustic trauma; D3, after sound conditioning and at 3 day post-acoustic trauma; D7, after sound conditioning and 7 day post-trauma; D14, after sound conditioning and at 14 day post-acoustic trauma.

Experimental animals were purchased from Spelford Biotechnology Company (Beijing, China). CBA male mice (12-weeks-old; weight, 28 ± 2 g) free of external or middle ear lesions, were used in this study. Animals were handled and treated according to the guidelines established by the Animal Care and Use Committee of the Chinese PLA General Hospital. Mice were randomly separated into the following groups (approximately 24 mice per group): Control, noise exposure (NE), 65 dB SPL sound conditioning plus noise exposure (65SC+NE), and 85 dB SPL sound conditioning plus noise exposure (85SC+NE). The study design is illustrated in Figure 1.

### Noise exposure

To elicit threshold shifts, awake mice in separate stainless steel wire cages (15 cm × 5 cm × 5 cm) were exposed to white noise at 110 dB SPL for 2 h. A loudspeaker (YH25-19B, 25 W, 16 W, China) powered by a power producer (33220A, China) fed by the noise program was used in the sound exposure chamber. Audio editing software was used to create and equalize noise sound software files. To verify the uniformity of sound fields, a sound level meter (Model 1200; Quest Technologies, Oconomovoc, WI, United States) was calibrated at multiple positions within the sound chamber. To guarantee consistency, sound levels were measured before and after each session.

For sound conditioning, the 65SC and 85SC groups were exposed to white noise at 65 or 85 dB SPL, respectively, for 8 h per day for 7 days. After resting for 24 h, both groups were exposed to 110 dB SPL white noise for 2 h.

### Auditory brainstem response threshold test

Time domain transmission (TDT) audiometry equipment (TDT, Alachua, Florida, United States) and Biosig audiometry software were used to test auditory brainstem response (ABR). Before audiometry, mice were anesthetized using 1% pentobarbital (1 mg/kg, intraperitoneal injection) and placed in a soundproof room. A recording electrode was subcutaneously implanted at the middle of the anterior margin of the auricle, and reference and recording electrodes were placed subcutaneously behind the ear. Stimulation sound level was gradually reduced in steps of 10 dB until it was no longer heard, starting at the maximum stimulation intensity (90 dB) and steadily reducing until no repetitive ABR waveform could be detected, then increased by 5 dB until a repetitive ABR waveform could be detected. ABR audiograms were performed before noise exposure, after sound conditioning and immediately (D0), and at 3 (D3), 7 (D7), and 14 (D14) days post-acoustic trauma for each individual.

In the present study, ABR wave I amplitudes and latencies evoked by 90 dB pure tone at 4, 8, and 16 kHz were collected and recorded for each group at D0 and D14 after sound conditioning. The 90 dB SPL sound that elicited wave I, as well as its initial negative (n) and subsequent positive (p) deflections, were measured. Ip–In (delay = 1.2–1.9 ms) was used to define wave I amplitude. MATLAB software was used to create an algorithm for automatically determining ABR amplitude (MathWorks, Natick, MA, United States).

### Cochlear basilar membrane processing

After completion of ABR testing, mice were sacrificed, and cochleae quickly removed from the skull, perfused with 4% paraformaldehyde, and fixed overnight through the round and oval windows and the apex. Cochlea shells were decalcified in 10% EDTA for 4–6 h after fixation, and the basal turn separated under a dissection microscope in 0.01 mmol/L PBS solution. Then, the vestibular and tectorial membranes were removed.

### Immunofluorescence staining

Isolated basilar membranes were punched in 0.5% Triton X-100 solution for 30 min, then blocked with 10% goat serum for 1 h at room temperature. Tissues were incubated overnight at 4°C with the following primary antibodies: monoclonal mouse anti-carboxyl-terminal binding protein 2 (CtBP2) (diluted 1:200; Abcam, Cambridge, United Kingdom); or monoclonal rabbit anti-AMPKα (T177) (diluted 1:50; Cell Signaling Technology, Boston, United States). After washing three times, specimens were incubated with secondary antibody (Alexa Fluor 568 or Alexa Fluor 488, 1:1,000, Thermo Fischer Scientific, Waltham, MA, United States) and phalloidin (1:1,000, Thermo Fischer Scientific, Waltham, MA, United States) for 1 h at room temperature. After a final wash, cochleae were mounted on slides and stained with DAPI (Abcam, Cambridge, United Kingdom). Prepared slides were inverted onto a Zeiss confocal microscope and observed under a 63× magnification oil microscope, with a scanning layer spacing of 0.35 μm/layer. Wavelengths of 405, 488, 555, and 647 nm were used for specimen laminar sweeping, corresponding to blue, green, orange, and red under the fluorescence laser, respectively. Laminar scan was initiated when the signal appeared and terminated when the signal faded, with all pictures overlapped to generate the final result. Mean fluorescence intensity values for relevant areas were determined using the Zeiss software measurement tool and the same sized area (approximately 60 outer hair cells) selected for each photograph.

### Protein extraction

One cochleae of each mouse was promptly extracted from skulls and dissected in PBS at 4°C to remove any excess tissue. Pooled tissue samples were lysed in RIPA sample buffer containing phosphatase inhibitor, protease inhibitor, and PMSF. To remove tissue debris, supernatants were centrifuged at 12,000 × g (Fresco17, Walthman, United States) for 20 min at 4°C, and protein concentrations determined using a Bio-Rad Protein Assay kit. Two cochleae from the same animal were combined for each sample.

### Western blot

Western blot analysis was used to evaluate levels of p-AMPK protein expression in mice. One cochleae of each mouse was quickly removed from temporal bones after decapitation. Under an anatomical microscope (Olympus, SZX7, Tokyo, Japan), soft tissues were extracted from cochleae and homogenized in RIPA lysis buffer. An enhanced BCA Protein Assay Kit was used to assess total protein concentrations. Aliquots (25 μg) of each protein lysate were separated by 12% SDS-PAGE and transferred to polyvinylidene difluoride membranes. After incubation at room temperature for 1 h in blocking solution (5% non-fat dried milk in tris-buffered saline containing 1% Tween-20 [TBST]), membranes were washed and dried. Subsequently, blots were incubated overnight with primary antibodies (1:500, p-AMPKα, Cell Signaling Technology, Boston, United States; 1:1,000, β-actin, Abcam, Cambridge, United Kingdom), washed five times for 5 min each with PBST, then incubated with appropriate secondary antibodies at 1:1,000 for 1 h at room temperature. Then, after the membranes were washed well, immunoreactive bands were observed by ECL. X-ray film was scanned and analyzed using Image J software, and background staining density of an area with no bands subtracted from band densities, then target protein/β-actin ratios were calculated to obtain the relative expression levels of target proteins. Finally, differences were statistically analyzed, using at least three samples per group.

### Statistical analysis

Statistical analyses were performed using GraphPad Prism 8 and SPSS 25.0 software. Data variability and the extent of differences between groups were used to calculate *in vivo* group sizes (n). One-way ANOVA with Tukey’s multiple comparisons, repeated-measures ANOVA with *post hoc* testing, unpaired *t*-tests, one-sample t tests, and linear regression analysis were among the statistical procedures applied. Multivariate ANOVA was used for analyses of ABR thresholds, amplitudes, and wave I latencies, while one-way ANOVA was used for analysis of synapse count, p-AMPKα fluorescence intensity, and protein expression, and linear regression analyses were used to assess p-AMPK integrated density and ribbon synapse numbers. All tests were two-tailed, and *p* < 0.05 was considered significant.

## Results

### Sound conditioning protects the auditory system and prevents noise-induced hearing loss

**FIGURE 2:**
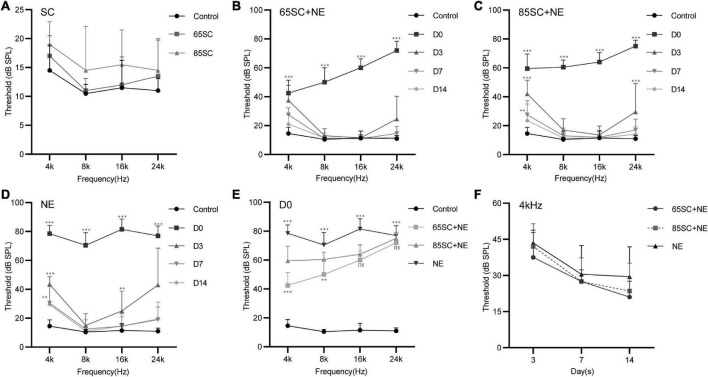
Hearing thresholds of mice after sound conditioning and changes in hearing thresholds of mice in each group after 110 dB SPL noise exposure. **(A)** ABR thresholds did not differ between SC groups and the control group after sound conditioning (*p* > 0.05). **(B)** Changes in ABR thresholds at different time points after noise exposure in the 65SC+NE group: the highest hearing threshold was at D0 (*p* < 0.001), and hearing thresholds returned to normal at D14 (*p* > 0.05). **(C)** Changes in ABR thresholds at various time points after noise exposure in the 85SC+NE group: the highest threshold was at D0 (*p* < 0.001), and hearing thresholds returned to normal at D14 (*p* > 0.05). **(D)** ABR thresholds in the NE group at each time point after noise exposure: the highest threshold was at D0 (*p* < 0.001), and hearing thresholds returned to normal at D14 except 4 kHz frequency (*p* > 0.05). **(E)** Comparison of ABR thresholds in the control and experimental groups at D0. Sound conditioning contributed to hearing protection immediately after noise exposure, and hearing protection in the 65SC+NE group was superior to that in the 85SC+NE group, primarily at 4 and 8 kHz frequencies. **(F)** The 65SC+NE, 85SC+NE, and NE groups were more seriously affected by noise at 4 kHz. **p < 0.01, ***p < 0.001.

Auditory brainstem response thresholds in the Control, SC, NE, and SC+NE groups were investigated at each time point (D0, D3, D7, and D14). The 65SC and 85SC groups did not differ significantly from the control group after sound conditioning (Figure 2A and Table 1).

**TABLE 1:** Auditory brainstem response (ABR) thresholds for each group at the end of the sound conditioning (dB SPL).

Group	Click	4 kHz	8 kHz	16 kHz	24 kHz
Control	13.00 ± 2.58	14.50 ± 4.38	10.50 ± 1.58	11.50 ± 4.74	11.00 ± 2.11
65SC	15.50 ± 4.97	17.00 ± 3.50	11.00 ± 2.11	12.00 ± 4.83	13.50 ± 6.26
85SC	20.00 ± 5.27	19.00 ± 3.94	14.50 ± 7.62	15.50 ± 5.99	14.50 ± 5.50
*P*	0.075	0.058	0.417	0.064	0.308

After exposure to 110 dB SPL white noise for 2 h, the hearing thresholds of all three experimental groups were considerably higher than those of the control group (*p* < 0.001). The ABR thresholds of the NE group were markedly higher than those of the 65SC+NE and 85SC+NE groups (*p* < 0.001). Further, ABR thresholds at Click, 4 and 8 kHz were significantly lower in the 65SC+NE group than those in the 85SC+NE group (*p* < 0.01, Figures 2B,C,E and Table 2).

**TABLE 2:** Auditory brainstem response (ABR) thresholds of each group at the moment of noise exposure (D0) (dB SPL).

Group	Click	4 kHz	8 kHz	16 kHz	24 kHz
Control	13.00 ± 2.58	14.50 ± 4.38	10.50 ± 1.58	11.50 ± 4.74	11.00 ± 2.11
65SC+NE	43.00 ± 12.06	42.50 ± 8.90	50.00 ± 10.00	60.00 ± 6.24	72.00 ± 6.32
85SC+NE	62.50 ± 7.17	59.50 ± 10.12	60.50 ± 4.97	64.00 ± 6.58	75.00 ± 4.08
NE	75.50 ± 8.32	78.50 ± 5.8	70.50 ± 8.64	81.50 ± 7.09	77.00 ± 6.75
*P*	<0.001***	<0.001***	<0.001***	<0.001***	<0.001***

***p < 0.001.

Following exposure to 110 dB SPL white noise, thresholds of the three groups gradually recovered on D3. The highest hearing thresholds were recorded at 4 and 24 kHz in all three animal groups. At 8 and 16 kHz, hearing thresholds in the 65SC+NE and 85SC+NE groups recovered to normal, while the NE group had significantly higher hearing thresholds than the SC+NE groups across all frequencies (*p* < 0.05) (Figures 2B–D,F and Table 3).

**TABLE 3:** Auditory brainstem response (ABR) thresholds for each group at the moment of noise exposure (D3) (dB SPL).

Group	Click	4 kHz	8 kHz	16 kHz	24 kHz
Control	13.00 ± 2.58	14.50 ± 4.38	10.50 ± 1.58	11.50 ± 4.74	11.00 ± 2.11
65SC+NE	22.00 ± 5.37	37.50 ± 10.34	13.00 ± 4.83	11.50 ± 2.42	24.50 ± 15.89
85SC+NE	21.50 ± 6.26	42.00 ± 9.49	17.00 ± 7.89	13.50 ± 6.26	29.50 ± 19.64
NE	23.00 ± 9.19	43.50 ± 5.3	15.00 ± 8.16	25.00 ± 13.74	43.00 ± 25.52
*P*	0.036*	<0.001***	0.201	0.014*	<0.001***

*p < 0.05, ***p < 0.001.

On D7 after exposure, the 4 kHz hearing thresholds of the three experimental groups remained significantly higher than those of the control group, while thresholds at 8, 16, and 24 kHz were no longer significantly different from those of the control group (*p* > 0.05). The hearing thresholds of the NE group at 4 kHz remained higher than that of the control group (*p* < 0.05). The ABR thresholds of SC+NE group had all returned to normal levels by D14 (Figures 2B–D,F).

In animals treated by sound conditioning, hearing thresholds recovered more quickly. Hence, our data show that sound conditioning reduced subsequent high-intensity noise-induced hearing damage, with the protective effect being most pronounced in the immediate aftermath of loud noise. Further, sound conditioning with 65 dB SPL was significantly more effective in protecting hearing than conventional sound conditioning with 85 dB SPL.

**FIGURE 3:**
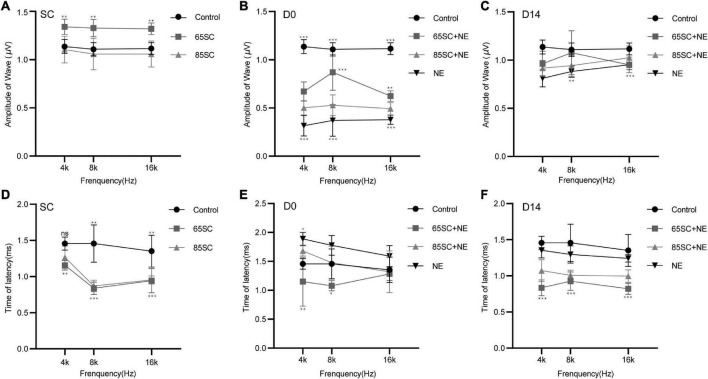
Amplitudes and Latencies of wave I at each time point after sound conditioning or noise exposure. **(A)** The amplitude of wave I in the 65SC group significantly increased than that in the control group at all frequencies after sound conditioning (*p* < 0.01); the amplitude of wave I did not differ between the 85SC group and the control group (*p* > 0.05). **(B)** ABR I wave amplitudes decreased significantly in all groups at D0. The amplitude of wave I in the NE group decreased obviously (*p* < 0.001). Comparing with 65SC+NE group, the amplitude of wave I in the 85SC+NE group was decreased at 8 kHz (*p* < 0.001) and 16 kHz (*p* < 0.01). **(C)** At D14, wave I recovered at all frequencies for all groups, with the 65SC+NE and 85SC+NE groups recovering to pre-exposure levels, while the NE group had not fully recovered at 8 kHz (*p* < 0.01) and 16 kHz (*p* < 0.001). **(D)** The latency of wave I in the 65SC group was significantly shorter than that of the control group at all frequencies after sound conditioning especially at 8 and 16 kHz (*p* < 0.001); and latency in the 85SC group was also shorter than that in the control group, except at 4 kHz (*p* < 0.01); the latency of wave I did not differ between the 65SC group and the 85SC group (*p* > 0.05). **(E)** The latency of wave I did not differ between the SC+NE groups and the control group at D0 (*p* > 0.05); and in the NE group the latency of wave I was prolonged especially at 4 kHz (*p* < 0.05). Comparing with 85SC+NE group, the latency of wave I in the 65SC+NE group was shorter at 4 kHz (*p* < 0.01) and 8 kHz (*p* < 0.05). **(F)** The latency of wave I did not differ between the NE group and the control group at D14 (*p* > 0.05); the latency of wave I in the SC+NE groups was still significantly shorter than that of the control group (*p* < 0.001). *p < 0.05, **p < 0.01, ***p < 0.001.

After sound conditioning, ABR I wave amplitude in the 65SC group was significantly higher than that in the control group (*p* < 0.01); amplitude did not differ between the 85SC group and the control group (*p* > 0.05) (Figure 3A). Immediately after noise exposure (D0), ABR I wave amplitude decreased significantly in all groups, with the most significant decrease in the NE group. Wave I amplitude in the 65SC+NE group was significantly higher than that in the 85SC+NE group at 8 kHz (*p* < 0.001) and 16 kHz (*p* < 0.01) (Figure 3B). Further, at D14 after noise exposure, wave I recovered at all frequencies for all groups, with the 65SC+NE and 85SC+NE groups recovering to pre-exposure levels, while the NE group had not fully recovered at 8 kHz (*p* < 0.01) and 16 kHz (*p* < 0.001) (Figure 3C).

Wave I latency in the 65SC group was significantly shorter than that in the control group especially at 8 and 16 kHz (*p* < 0.001), and latency in the 85SC group was also shorter than that in the control group, except at 4 kHz (*p* < 0.01) (Figure 3D). At D0, latency was prolonged in the NE group at 4 kHz compared with the control group (*p* < 0.05). In contrast, there was no significant difference between SC+NE groups and the control group (*p* > 0.05) (Figure 3E). At D14, latency was reduced in the NE group, while it remained significantly shorter in the SC+NE groups compared with the control group (*p* < 0.001) (Figure 3F).

### Sound conditioning activates adenylate activated kinase, resulting in increased expression of p-AMPKα in the cochlea

**FIGURE 4:**
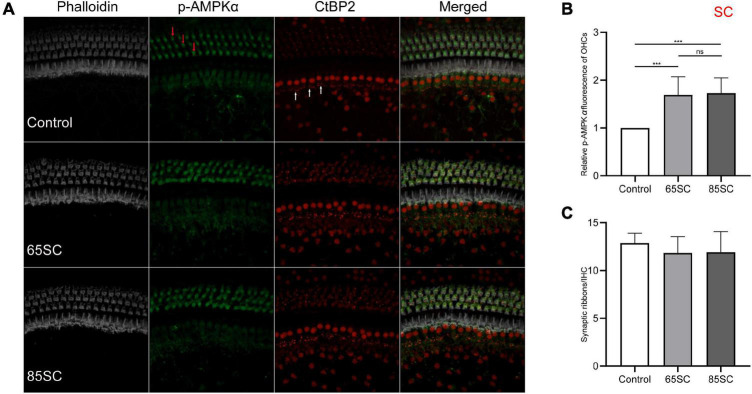
Sound conditioning activated AMPK, and did not decrease ribbon synapses. **(A)** p-AMPKα fluorescence intensity in outer hair cells (red arrows) was increased relative to the unexposed control group on exposure to 65 or 85 dB SPL white noise for 1 week; CtBP2 (white arrows) was not significantly reduced. **(B)** Quantification of p-AMPKα immunolabeling indicating that sound conditioning may activate AMPK. There was no significant difference in p-AMPKα fluorescence intensity between the 65SC and 85SC groups. **(C)** Ribbon synaptic amount did not differ significantly between sound conditioning and control group animals. ***p < 0.001.

To investigate AMPK activation in the presence of noise, we used immunolabeling and western blotting analyses to assess p-AMPKα expression in cochleae from each group of mice. The fluorescence intensity of p-AMPKα increased in the SC groups compared to the control group (*p* < 0.001), and the results of western blotting revealed that p-AMPK levels increased following sound conditioning, with protein levels in the 65SC group was twice that of the control group, and in the 85SC group 2.7 times higher than those of the control group (Figures 4, 9A,A’).

**FIGURE 5:**
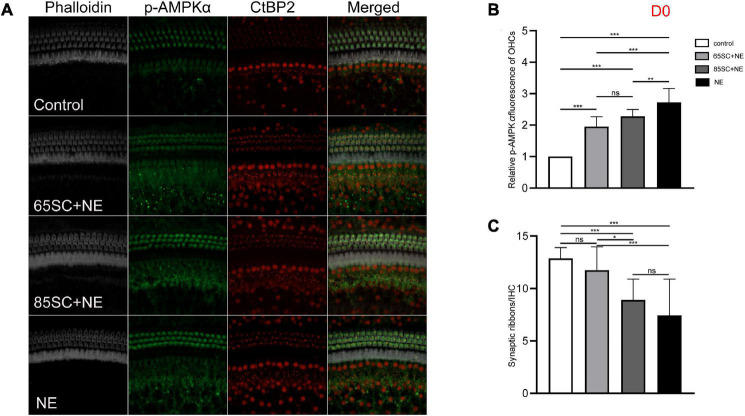
The p-AMPKα fluorescence intensity and the number of ribbon synapses changed in each group of mice after being exposed to 110 dB SPL noise at D0. **(A)** The p-AMPKα fluorescence intensity and the number of synapses of the four groups at D0. **(B)** NE group showed the highest p-AMPKα fluorescence intensity (*p* < 0.001), and the intensity of SC+NE groups were stronger than the control group (*p* < 0.001). **(C)** The number of synapses did not differ between the 65SC+NE group and the control group (*p* > 0.05), and both the 85SC+NE group and the NE group showed less synapses than the control group and 65 SC group. *p < 0.05, **p < 0.01, ***p < 0.001.

**FIGURE 6:**
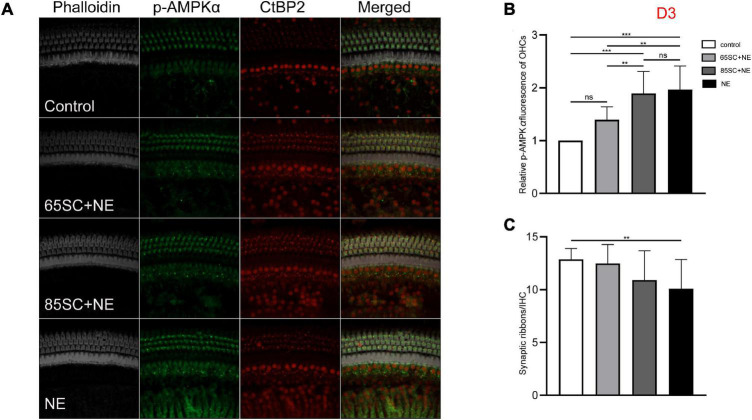
The p-AMPKα fluorescence intensity and the number of ribbon synapses restored in all groups at D3. **(A)** The p-AMPKα fluorescence intensity and the number of synapses of the four groups at D3. **(B)** The p-AMPKα fluorescence intensity in the four groups from the highest to the lowest was in the NE group, 85SC+NE group, 65SC+NE group, and control group. Among them, there was no statistical difference between the 65SC+NE group and the control group (*p* > 0.05). **(C)** The number of synapses in the NE group was lower than the control group (*p* < 0.01), and the number of synapses did not differ between the SC+NE groups and the control group (*p* > 0.05). **p < 0.01, ***p < 0.001.

**FIGURE 7:**
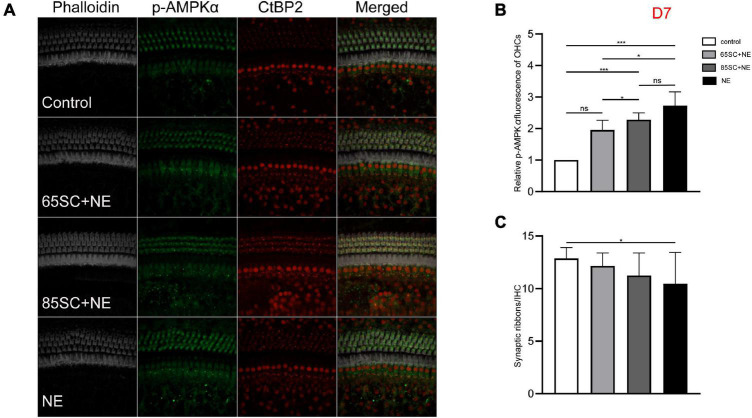
The p-AMPKα fluorescence intensity decreased and the number of ribbon synapses gradually recovered in the noise exposed groups at D7. **(A)** The p-AMPKα fluorescence intensity and the number of synapses of the four groups at D7. **(B)** There was no significant difference in p-AMPKα fluorescence intensity between the 65SC+NE group and the control group (*p* > 0.05), p-AMPKα fluorescence intensity of the NE and 85SC+NE groups was higher than of the control group (*p* < 0.001), the NE group showed greater p-AMPKα fluorescence intensity than the 65SC+NE group (*p* < 0.05). **(C)** Among the four groups, the NE group had the least number of ribbon synapses (*p* < 0.05). *p < 0.05, ***p < 0.001.

**FIGURE 8:**
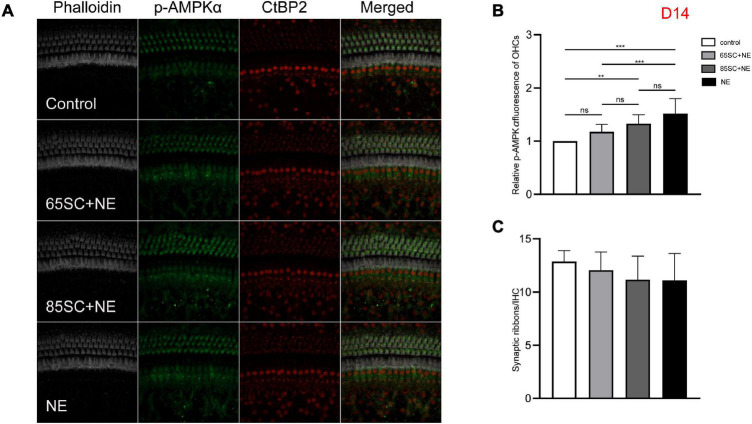
The p-AMPKα fluorescence intensity and the number of ribbon synapses returned to normal at D14. **(A)** The p-AMPKα fluorescence intensity and the number of synapses of the four groups at D14. **(B)** The p-AMPKα fluorescence intensity of the NE group was higher than that of the control (*p* < 0.001) and the 65SC+NE group (*p* < 0.01), the p-AMPKα fluorescence intensity did not differ between the 65SC+NE group and the 85SC+NE group (*p* > 0.05). **(C)** The number of synapses did not differ between the noise exposed animals and the controls (*p* > 0.05). **p < 0.01, ***p < 0.001.

**FIGURE 9:**
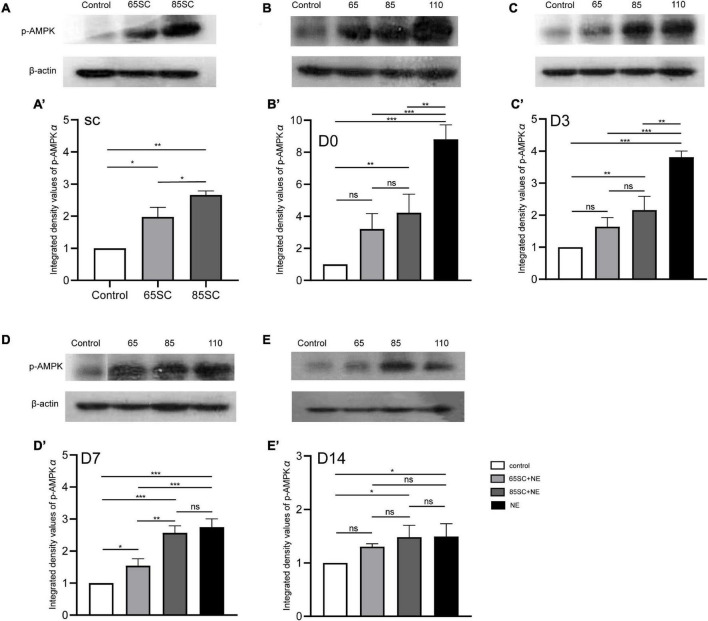
The expression of the p-AMPK protein in the cochlea of mice varied in each group at different time point. **(A,A’)** p-AMPK protein expression increased in the SC groups after sound conditioning, and the 85SC group showed higher expression than the 65SC group (*p* < 0.05). **(B,B’)** Among the four groups, it showed the highest p-AMPK protein expression in the NE group (*p* < 0.001), followed by the 85SC+NE group at D0 (*p* < 0.01); there was no statistical difference between the 65SC+NE group and the control group (*p* > 0.05); and the NE group’s protein gray value was significantly higher than the 65SC+NE group (*p* < 0.001) and the 85SC+NE group (*p* < 0.01). **(C**,**C’)** The expression of p-AMPK protein reduced in each experimental group at D3. **(D**,**D’)** The p-AMPK protein level in the NE group decreased obviously at D7, but there was no significant change when comparing to the 85SC+NE group (*p* > 0.05). **(E**,**E’)** The p-AMPK protein expression in the 85SC+NE group and NE group were higher than the control group at D14 (*p* < 0.05). *p < 0.05, **p < 0.01 ***p < 0.001.

At D0, all groups, particularly the NE group, had increased p-AMPK expression, with fluorescence intensity values 2.7 times higher than those of the control group. Protein band gray scale values also differed significantly at approximately 9-fold those of the control group (*p* < 0.001). Fluorescence intensity values in the 65SC+NE and 85SC+NE groups did not differ significantly from one another (*p* > 0.05), and were twice that of the control group (*p* < 0.001). Protein gray scale values in the 65SC+NE group did not differ significantly from those of the control group (*p* > 0.05), but were approximately 4.2 times higher and significantly different in the 85SC+NE group compared to the control group (*p* < 0.01), around 2.8 times higher in the NE group than the 65SC+NE group (*p* < 0.001), and 2.1 times higher (*p* < 0.01) than the 85SC+NE group (Figures 5, 9B,B’).

At D3, the p-AMPKα fluorescence intensity and protein gray scale values of the 65SC+NE group were no longer significantly different from those of the control group (*p* > 0.05); the fluorescence intensity of the NE group was almost twice that of the control group, while protein band gray scale values were 3.8 times that of the control group (*p* < 0.001); the fluorescence intensity of the 85SC+NE group was 2.2 times that of the control group (*p* < 0.001), and the protein gray scale value was 1.9 times higher than that of the control group (*p* < 0.01). Fluorescence intensity of p-AMPKα did not differ significantly between the NE and 85SC+NE groups (*p* > 0.05), while protein gray scale values of the NE group were about 2 times higher than that of the 85SC+NE group (*p* < 0.01). Further, fluorescence intensity of the NE group was 1.5 times greater than that of the 65SC+NE group (*p* < 0.001), and protein expression was 2.3 times higher than that of the control group (*p* < 0.001). Furthermore, fluorescence intensity of the 85SC+NE group was 1.4 times that of the 65SC+NE group (*p* < 0.01), while there was no significant difference in protein gray scale value (*p* > 0.05) (Figures 6, 9C,C’).

At D7, the NE and 85SC+NE groups showed significant changes in p-AMPKα fluorescence intensity and protein gray scale values relative to the control group (*p* < 0.001), but the p-AMPKα fluorescence intensity and protein gray scale values did not differ between the 65SC+NE group and the control group (*p* > 0.05). Fluorescence intensity in the NE group was 1.2 times that of the 65SC+NE group (*p* < 0.05), while protein expression was twice that in the control group (*p* < 0.001). Compared with the 65SC+NE group, 85SC+NE fluorescence intensity was 1.25 times (*p* < 0.05) and gray scale value 1.7 times (*p* < 0.01), which were significant differences. At this time point, p-AMPKα fluorescence intensity in the 65SC+NE group did not differ significantly from that in controls (*p* > 0.05), while protein expression was 1.5 times higher, which was significantly different (*p* < 0.05) (Figures 7, 9D,D’).

At D14, fluorescence intensity in the 85SC+NE group was 1.5 times that of the control group (*p* < 0.01), while that of the NE group was 1.7 times higher (*p* < 0.001); the protein gray scale values of the NE and 85SC+NE groups did not differ significantly from one another (*p* > 0.05), but were slightly higher than that of the control group (*p* < 0.05). There was no significant change in fluorescence intensity or protein expression in the 65SC+NE group relative to the 85SC+NE group (Figures 8, 9E,E’).

### Sound conditioning protects synapses from high intensity noise damage

The number of synapses in each specimen was counted individually in IHCs, and the differences between the control, SC, SC+NE, and NE groups assessed at each time point.

Mice in the 65 and 85 dB SPL condition groups had 11.84 **±** 1.72 and 11.91 **±** 2.15 synapses per IHC after 1 week of sound conditioning, which was not significantly different from the control group (12.88 **±** 1.03; *p* > 0.05) (Figures 4A,B).

After 110 dB SPL noise exposure (D0), mean numbers of synapses in the NE (7.43 **±** 3.47) and 85SC+NE group (9.47 **±** 1.21) groups were significantly reduced, and differed significantly from the control group (*p* < 0.001). Mean number of synapses in the 65SC+NE group (11.2 **±** 42.14) did not differ significantly from the control group (*p* > 0.05), but were significantly higher than in the NE group (*p* < 0.001). The 65SC+NE group had significantly more synapses than the 85SC+NE group (*p* < 0.05) (Figures 5A,B).

The number of synapses in the 85SC+NE group had recovered to normal at D3 after noise, but there remained a significant difference between numbers of synapses in the NE and control groups until D14 (*p* < 0.001) (Figure 6), when the number of synapses in the NE group recovered completely, and there was no significant difference between the groups (p > 0.05) (Figures 7, 8).

### Activation of adenylate activated kinase did not correspond with changes in synapse number during low-intensity sound conditioning, while activated p-AMPK content and synapse number were negatively correlated during high-intensity stimulation

Adenylate activated kinase was activated and p-AMPK expression enhanced when low-intensity noise was delivered; however, the number of ribbon synapses did not change appreciably relative to the control group. At D0, the SC+NE groups had more ribbon synapses than the NE group, despite p-AMPK expression being lower in the SC+NE groups. The 65SC+NE group had the lowest p-AMPK fluorescence intensity and the most synapses, while the NE group had the highest p-AMPK fluorescence intensity and the fewest synapses, at D3, D7, and D14 (Figure 4).

### After noise exposure, changes in p-AMPK and ribbon synapses in each group of animals were negatively correlated

**FIGURE 10:**
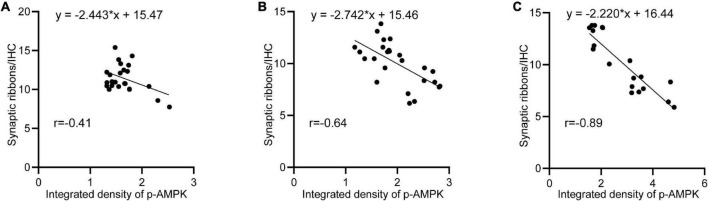
Negative correlation between p-AMPK and the amount of ribbon synapses. **(A)** Linear regression analysis of p-AMPK intensity and the number of ribbon synapses at D0, D3, D7, and D14 in the 65SC and 65SC+NE groups (*r* = –0.41, *p* < 0.05). **(B)** Linear regression analysis of p-AMPK intensity versus the number of ribbon synapses at D0, D3, D7, and D14 in the 85SC and 85SC+NE groups (*r* = –0.84, *p* < 0.05). **(C)** Linear regression analysis of p-AMPK intensity versus the number of ribbon synapses at D0, D3, D7, and D14 in the NE group (*r* = –0.89, *p* < 0.001).

After noise exposure, p-AMPK intensity changed in all groups of animals, with the lowest intensity in the 65SC group after sound conditioning and the highest intensity at D0 in the NE group. In contrast, the number of ribbon synapses showed the opposite trend in all groups, and correlation analysis detected a negative correlation between p-AMPK intensity and the number of ribbon synapses (Figure 10).

## Discussion

### Sound conditioning protects the auditory system from noise-induced hearing loss, while enabling faster recovery from transient threshold shift

Canlon et al. (1988) was the first to describe sound conditioning, using an 81 dB SPL, 1 kHz sound stimulus for conditioning. Sound conditioning has since been reported to reduce noise-induced hearing loss. Although the precise mechanism involved is not completely understood, the underlying physiological adaptation responses may be as follows: 1. Sound conditioning increases the movement capacity of outer hair cells, as well as their aptitude to adapt to repeated environmental stimulation and post-stimulation fatigue, which enables the hair cells to actively screen or reduce persistent auditory stimulations, as a protective mechanism, at the ipsilateral cochlea and basilar membrane level; 2. Sound conditioning may downregulate glucocorticoid receptor expression induced by trauma, which may also protect the central components of the HPA; 3. Sound conditioning prevents reorganization of the cortical tonotopic map in cats after cochlear damage, which suggests a positive effect on both the central and peripheral auditory systems; 4. Sound conditioning increases levels of antioxidant enzymes in the cochlea, thereby enhancing free radical scavenging and protecting the organ of Corti from noise-induced damage by increasing stria vascularis levels of catalase, a hydrogen-peroxide-scavenging enzyme (Hu et al., 1997; Yamasoba et al., 1999); and 5. Induction of high heat shock protein (HSP) expression levels by sound conditioning have been demonstrated (Dechesne et al., 1992; Thompson and Neely, 1992). When HSP levels are high, animals show greater recovery from noise trauma relative to those without high HSP levels (Myers et al., 1992). There are also other theories about the underlying mechanisms, such as upgrading of calcium-buffering systems and increases in some neurotrophic factors, among others (Niu and Canlon, 2002). Regardless of which mechanism is most dominant, they will ultimately cause metabolic changes leading to “toughening” or resistance to noise-induced hearing loss. As demonstrated in this study, when mice underwent conditioning for 1 week, wave I amplitude, which indicates the firing potential at the site of connection between IHCs and type I spiral ganglion neurons (Moser et al., 2020), and represents synchronous evoked activity of cochlear nerve fibers in response to acoustic stimulation, was increased, indicating an active function of connections among IHCs after noise exposure. Further, conditioned animals showed mild hearing loss and faster recovery than controls.

### The lowest sound intensity that induced sound conditioning was 65 dB sound pressure level

Several animal studies with noise levels ranging from 85 to 100 dB SPL, have been conducted to evaluate sound conditioning settings (Campo et al., 1991; Subramaniam et al., 1992; Dagli and Canlon, 1997). Yoshida et al. devised two sound conditioning techniques using CBA male mice: (1) Sound conditioning at 81 dB SPL, 8.0–16.0 kHz, for 1 week; and (2) 15 min of 89 dB SPL, 8.0–16.0 kHz sound conditioning, followed by 2 h exposure to the same frequency of high-intensity noise at 100 dB SPL. The experiments revealed that both sound conditioning plus noise exposure groups exhibited significantly reduced subsequent severe noise-induced compound action potential (CAP) and distortion product otoacoustic emissions (DPOAE) threshold shifts (Yoshida and Liberman, 2000).

Sheppard et al. (2018) employed a lower-intensity experimental setting, in which rats were exposed to noise at 65 dB SPL, 10–20 kHz for 5 weeks, and found no significant increase in DPOAE, but a significant increase in CAP amplitude, after a week of rest, relative to the control group. Further, after 6 weeks exposure to low-intensity noise at 18–24 kHz, 55 dB SPL, Liu et al. (2020) showed a reduction in CAP amplitude.

Discrepancies in experimental design, such as experimental animal selection and characteristics, including the frequency spectrum, sound level, and period of sound conditioning and noise blast, can easily lead to contradictory outcomes.

The sound conditioning conditions used in this experiment were 65 and 85 dB SPL white noise. White noise at 65 dB SPL, which is the lowest noise level known to cause habituation, can provide substantial protection. Relative to 85 dB SPL, 65 dB noise had a stronger beneficial effect on auditory protection, which was particularly clear in the immediate aftermath of loud noise and during the recovery period.

Mice exposed to narrow-band noise at 100 dB SPL for 2 h showed a transient increase in ABR thresholds, and when removed from the noise environment for 2 weeks, these animals showed a temporary hearing threshold shift, as ABR thresholds returned to pre-exposure levels (Kujawa and Liberman, 2009); this hypothesis has been supported by a number of studies (Liu et al., 2012; Shi et al., 2015). ABR thresholds recovered to pre-noise levels in both groups 2 weeks after high intensity noise, with the sound conditioning group recovering considerably faster than the non-sound conditioning group.

### Sound conditioning activates adenylate activated kinase to protect ribbon synapses from noise damage

Normal function of the cochlear ribbon synapse, which is positioned between the IHC and type I spiral ganglia, is critical for hearing conduction. The transit, aggregation, and release of cochlear ribbon synaptic presynaptic vesicles are all dependent on the consumption of massive amounts of ATP supplied by IHC mitochondria (Griesinger et al., 2005; Matthews and Fuchs, 2010). Consequently, the ability of mitochondria to produce enough ATP is critical for maintaining synaptic function. Strong noise exposure causes a high inward flow of calcium ions, which facilitates mitochondria-related cell death; calcium inward flow is linked to energy expenditure, which impairs mitochondrial metabolism and thus leads to apoptosis.

Oxidative stress plays an important role in the process of noise-induced hearing loss, as excessive production of reactive oxygen species can directly damage DNA, especially mitochondrial DNA (mt DNA), break C-H bonds in DNA pentose sugars, and break down nucleotides. Simultaneously, ROS can mediate direct peroxidation of unsaturated fatty acids on biological membranes and damage mitochondrial membranes, causing impaired energy metabolism (Caston and Demple, 2017). Downstream of ROS, inner ear stress-activated MAPKs (including JNK and p-AMPK) mediate cellular stress responses and inflammation through intermediate signaling protein activation (Wu F. et al., 2020), which in turn affects cell proliferation, differentiation, and apoptosis. Under hypoxic conditions in the inner ear after noise exposure, AMPK activates a ROS-dependent pathway and mediates outer hair cell apoptosis (Hill et al., 2016).

Adenylate activated kinase is a trimeric complex that is involved in cellular energy balance regulation. It is activated by noise-induced ATP decreases, leading to phosphorylation of downstream sites and restoration of energy balance by increasing catabolism (ATP generation) and decreasing anabolism (ATP utilization) (Housley et al., 2013; Carling, 2017). During loud noise exposure, a significant drop in ATP reduces the number of ribbon synapses. AMPK activation can function to adjust an organism to oxidative stress, while also inhibiting the cytotoxic response induced by glutamate. According to Hill et al. (2016) AMPK activation alone does not protect the inner ear from noise damage; instead, when p-AMPK accumulates to a certain level, it activates the JNK pathway and triggers apoptosis. These researchers found that CBA mice exposed to 98 and 106 dB SPL for hours had increased p-AMPK expression, but that ribbon synaptic release was much lower in the 106 dB SPL group than in the 98 dB group. They hypothesized that p-AMPK expression was noise-dependent, and used siAMPKa1 to block the AMPK activation site, revealing that a 30% drop in AMPK activation resulted in an 80% reduction in hearing loss (Hill et al., 2016). The findings of the current study also imply that sound conditioning may protect hearing *via* AMPK-mediated mechanisms, as follows: 1. Early activation of AMPK increases ATP reserves, which protects hair cells from death and synaptic loss caused by rapid ATP depletion during subsequent intense noise exposure. The modest dose of p-AMPK elicited by low-dose noise is insufficient to produce damage to the inner ear, which explains why 65 dB SPL sound conditioning is superior to 85 dB SPL sound conditioning. 2. As AMPK is a protein kinase, its activation is limited, and when sound conditioning causes a reduction in AMPK content or receptor, following activation of a proportion of AMPK, it cannot be activated as much as in normal animals when exposed to subsequent noise, but instead mitigates p-AMPK elevation. 3. Since AMPK activation can inhibit the cytotoxic response induced by glutamate, sound conditioning can protect inner hair ribbon synapses to mitigate the damage caused by subsequent noise-induced glutamate release.

One limitation of this study is that the role of upstream and downstream of AMPK pathway components in the sound conditioning protective mechanism was not thoroughly investigated. Further, we do not provide direct proof of the relationship between AMPK and ribbon synaptic release. As previously stated, a specific degree of activity of AMPK protects the inner ear, but once the activation product, p-AMPK, exceeds a specific concentration, it causes cochlear hair cell death and hearing disability. Future research work will aim to determine the “turning point” between cochlea protection and cochlea damage caused by AMPK activation and elucidate the role of AMPK in noise-induced deafness using antagonists.

By exposing CBA mice to low-intensity noise, we discovered that sound conditioning can activate AMPK and protect ribbon synapses from subsequent intense noise damage. We suspect that AMPK may restore some energy during sound conditioning, allowing ATP to be compensation and lowering ATP consumption under high noise. At varying noise intensities, however, the amount of AMPK activated will alter as p-AMPK levels change, resulting in diverse outcomes. This research adds to understanding of how sound conditioning protects hearing and implies that suitable experimental circumstances have potential for application in preventing hair cell loss and cochlear synaptopathy.

## Data availability statement

The original contributions presented in this study are included in the article/supplementary material, further inquiries can be directed to the corresponding authors.

## Ethics statement

The animal study was reviewed and approved by the Animal Care and Use Committee of Chinese PLA General Hospital.

## Author contributions

RZ participated in the analysis and interpretation of the data. LS and NY made substantial contributions to the conception and design of the study. CM and MW performed the statistical analysis. XL and WL contributed to the acquisition of data. All authors have read and approved the final manuscript.

## References

[B1] Alvarado J. C. Fuentes-Santamaria V. Gabaldon-Ull M. C. Jareno-Flores T. Miller J. M. Juiz J. M. (2016). Noise-induced “toughening” effect in wistar rats: enhanced auditory brainstem responses are related to calretinin and nitric oxide synthase upregulation. *Front. Neuroanat.* 10:19. 10.3389/fnana.2016.00019 27065815 PMC4815363

[B2] Campo P. Subramaniam M. Henderson D. (1991). The effect of ‘conditioning’ exposures on hearing loss from traumatic exposure. *Hear Res.* 55 195–200. 10.1016/0378-5955(91)90104-H 1757287

[B3] Canlon B. (1997). Protection against noise trauma by sound conditioning. *Ear. Nose Throat. J.* 76 253–245. 10.1177/014556139707600413 9127524

[B4] Canlon B. Borg E. Flock A. (1988). Protection against noise trauma by pre-exposure to a low level acoustic stimulus. *Hear Res.* 34 197–200. 10.1016/0378-5955(88)90107-4 3170362

[B5] Carling D. (2017). AMPK signalling in health and disease. *Curr. Opin. Cell Biol.* 45 31–37. 10.1016/j.ceb.2017.01.005 28232179

[B6] Caston R. A. Demple B. (2017). Risky repair: DNA-protein crosslinks formed by mitochondrial base excision DNA repair enzymes acting on free radical lesions. *Free Radic Biol. Med.* 107 146–150. 10.1016/j.freeradbiomed.2016.11.025 27867099 PMC5815828

[B7] Chen G. D. Decker B. Krishnan Muthaiah V. P. Sheppard A. Salvi R. (2014). Prolonged noise exposure-induced auditory threshold shifts in rats. *Hear Res.* 317 1–8. 10.1016/j.heares.2014.08.004 25219503 PMC4252814

[B8] Cody A. R. Johnstone B. M. (1982). Temporary threshold shift modified by binaural acoustic stimulation. *Hear Res.* 6 199–205. 10.1016/0378-5955(82)90054-5 7061351

[B9] Dagli S. Canlon B. (1997). The effect of repeated daily noise exposure on sound-conditioned and unconditioned guinea pigs. *Hear Res.* 104 39–46. 10.1016/s0378-5955(96)00179-7 9119765

[B10] Dechesne C. J. Kim H. N. Nowak T. S. Jr. Wenthold R. J. (1992). Expression of heat shock protein, HSP72, in the guinea pig and rat cochlea after hyperthermia: Immunochemical and in situ hybridization analysis. *Hear Res.* 59 195–204. 10.1016/0378-5955(92)90116-5 1618710

[B11] Fan L. Zhang Z. Wang H. Li C. Xing Y. Yin S. (2020). Pre-exposure to lower-level noise mitigates cochlear synaptic loss induced by high-level noise. *Front. Syst. Neurosci.* 14:25. 10.3389/fnsys.2020.00025 32477075 PMC7235317

[B12] Griesinger C. B. Richards C. D. Ashmore J. F. (2005). Fast vesicle replenishment allows indefatigable signalling at the first auditory synapse. *Nature* 435 212–215. 10.1038/nature03567 15829919

[B13] Harris K. C. Bielefeld E. Hu B. H. Henderson D. (2006). Increased resistance to free radical damage induced by low-level sound conditioning. *Hear Res.* 213 118–129. 10.1016/j.heares.2005.11.012 16466871

[B14] Henderson D. Bielefeld E. C. Harris K. C. Hu B. H. (2006). The role of oxidative stress in noise-induced hearing loss. *Ear Hear* 27 1–19. 10.1097/01.aud.0000191942.36672.f3 16446561

[B15] Hill K. Yuan H. Wang X. Sha S. H. (2016). Noise-induced loss of hair cells and cochlear synaptopathy are mediated by the activation of AMPK. *J. Neurosci.* 36 7497–7510. 10.1523/JNEUROSCI.0782-16.2016 27413159 PMC4945669

[B16] Housley G. D. Morton-Jones R. Vlajkovic S. M. Telang R. S. Paramananthasivam V. Tadros S. F. (2013). ATP-gated ion channels mediate adaptation to elevated sound levels. *Proc. Natl. Acad. Sci. U.S.A.* 110 7494–7499. 10.1073/pnas.1222295110 23592720 PMC3645545

[B17] Hu B. H. Zheng X. Y. Mcfadden S. L. Kopke R. D. Henderson D. (1997). R-phenylisopropyladenosine attenuates noise-induced hearing loss in the chinchilla. *Hear Res.* 113 198–206. 10.1016/s0378-5955(97)00143-3 9387999

[B18] Kapoor N. Mani K. V. Shukla M. (2019). Distortion product oto-acoustic emission: A superior tool for hearing assessment than pure tone audiometry. *Noise Health* 21 164–168. 10.4103/nah.NAH_37_19 32719303 PMC7650854

[B19] Kujawa S. G. Liberman M. C. (2009). Adding insult to injury: Cochlear nerve degeneration after “temporary” noise-induced hearing loss. *J. Neurosci.* 29 14077–14085. 10.1523/JNEUROSCI.2845-09.2009 19906956 PMC2812055

[B20] Kurabi A. Keithley E. M. Housley G. D. Ryan A. F. Wong A. C. (2017). Cellular mechanisms of noise-induced hearing loss. *Hear Res.* 349 129–137. 10.1016/j.heares.2016.11.013 27916698 PMC6750278

[B21] Liu L. Wang H. Shi L. Almuklass A. He T. Aiken S. (2012). Silent damage of noise on cochlear afferent innervation in guinea pigs and the impact on temporal processing. *PLoS One* 7:e49550. 10.1371/journal.pone.0049550 23185359 PMC3504112

[B22] Liu X. Li L. Chen G. D. Salvi R. (2020). How low must you go? Effects of low-level noise on cochlear neural response. *Hear Res.* 392:107980. 10.1016/j.heares.2020.107980 32447098

[B23] Liu Y. Tang G. Li Y. Wang Y. Chen X. Gu X. (2014). Metformin attenuates blood-brain barrier disruption in mice following middle cerebral artery occlusion. *J. Neuroinflam.* 11:177. 10.1186/s12974-014-0177-4 25315906 PMC4201919

[B24] Maison S. F. Usubuchi H. Liberman M. C. (2013). Efferent feedback minimizes cochlear neuropathy from moderate noise exposure. *J. Neurosci.* 33 5542–5552. 10.1523/JNEUROSCI.5027-12.2013 23536069 PMC3640841

[B25] Matthews G. Fuchs P. (2010). The diverse roles of ribbon synapses in sensory neurotransmission. *Nat. Rev. Neurosci.* 11 812–822. 10.1038/nrn2924 21045860 PMC3065184

[B26] Moser T. Grabner C. P. Schmitz F. (2020). Sensory processing at ribbon synapses in the retina and the cochlea. *Physiol. Rev.* 100 103–144. 10.1152/physrev.00026.2018 31373863

[B27] Myers M. W. Quirk W. S. Rizk S. S. Miller J. M. Altschuler R. A. (1992). Expression of the major mammalian stress protein in the rat cochlea following transient ischemia. *Laryngoscope* 102 981–987. 10.1288/00005537-199209000-00005 1518362

[B28] Niu X. Canlon B. (2002). Protective mechanisms of sound conditioning. *Adv. Otorhinolaryngol.* 59 96–105. 10.1159/000059246 11885667

[B29] Niu X. Shao R. Canlon B. (2003). Suppression of apoptosis occurs in the cochlea by sound conditioning. *Neuroreport* 14 1025–1029. 10.1097/01.wnr.0000070830.57864.32 12802196

[B30] Pukkila M. Zhai S. Virkkala J. Pirvola U. Ylikoski J. (1997). The “toughening” phenomenon in rat’s auditory organ. *Acta Otolaryngol. Suppl.* 529 59–62. 10.3109/00016489709124081 9288269

[B31] Qiu J. Wang M. Zhang J. Cai Q. Lu D. Li Y. (2016). The neuroprotection of sinomenine against ischemic stroke in mice by suppressing NLRP3 inflammasome via AMPK signaling. *Int. Immunopharmacol.* 40 492–500. 10.1016/j.intimp.2016.09.024 27769021

[B32] Roy S. Ryals M. M. Van Den Bruele A. B. Fitzgerald T. S. Cunningham L. L. (2013). Sound preconditioning therapy inhibits ototoxic hearing loss in mice. *J. Clin. Invest.* 123 4945–4949. 10.1172/JCI71353 24216513 PMC3809804

[B33] Sheppard A. Liu X. Ding D. Salvi R. (2018). Auditory central gain compensates for changes in cochlear output after prolonged low-level noise exposure. *Neurosci. Lett.* 687 183–188. 10.1016/j.neulet.2018.09.054 30273699 PMC6383362

[B34] Shi L. Liu K. Wang H. Zhang Y. Hong Z. Wang M. (2015). Noise induced reversible changes of cochlear ribbon synapses contribute to temporary hearing loss in mice. *Acta Otolaryngol.* 135 1093–1102. 26139555 10.3109/00016489.2015.1061699

[B35] Subramaniam M. Henderson D. Campo P. Spongr V. (1992). The effect of ‘conditioning’ on hearing loss from a high frequency traumatic exposure. *Hear Res.* 58 57–62. 1559906 10.1016/0378-5955(92)90008-b

[B36] Suryadevara A. C. Wanamaker H. H. Pack A. (2009). The effects of sound conditioning on gentamicin-induced vestibulocochlear toxicity in gerbils. *Laryngoscope* 119 1166–1170. 10.1002/lary.20145 19301415

[B37] Tahera Y. Meltser I. Johansson P. Salman H. Canlon B. (2007). Sound conditioning protects hearing by activating the hypothalamic-pituitary-adrenal axis. *Neurobiol. Dis.* 25 189–197. 10.1016/j.nbd.2006.09.004 17056263

[B38] Thompson A. M. Neely J. G. (1992). Induction of heat shock protein in interdental cells by hyperthermia. *Otolaryngol. Head Neck Surg.* 107 769–774. 10.1177/019459988910700611.1 1470456

[B39] Waqas M. Gao S. Iram Us S. Ali M. K. Ma Y. Li W. (2018). Inner ear hair cell protection in mammals against the noise-induced cochlear damage. *Neural Plast* 2018:3170801. 30123244 10.1155/2018/3170801 PMC6079343

[B40] Weisova P. Davila D. Tuffy L. P. Ward M. W. Concannon C. G. Prehn J. H. (2011). Role of 5′-adenosine monophosphate-activated protein kinase in cell survival and death responses in neurons. *Antioxid Redox Sign.* 14 1863–1876. 10.1089/ars.2010.3544 20712420

[B41] Winder W. W. Thomson D. M. (2007). Cellular energy sensing and signaling by AMP-activated protein kinase. *Cell Biochem. Biophys* 47 332–347. 17652779 10.1007/s12013-007-0008-7

[B42] Wu F. Xiong H. Sha S. (2020). Noise-induced loss of sensory hair cells is mediated by ROS/AMPKalpha pathway. *Redox Biol* .29:101406. 10.1016/j.redox.2019.101406 31926629 PMC6933152

[B43] Wu J. Ye J. Kong W. Zhang S. Zheng Y. (2020). Programmed cell death pathways in hearing loss: A review of apoptosis, autophagy and programmed necrosis. *Cell Prolif.* 53:e12915. 33047870 10.1111/cpr.12915 PMC7653260

[B44] Xue B. Kahn B. B. (2006). AMPK integrates nutrient and hormonal signals to regulate food intake and energy balance through effects in the hypothalamus and peripheral tissues. *J. Physiol.* 574 73–83. 10.1113/jphysiol.2006.113217 16709629 PMC1817809

[B45] Yamasoba T. Schacht J. Shoji F. Miller J. M. (1999). Attenuation of cochlear damage from noise trauma by an iron chelator, a free radical scavenger and glial cell line-derived neurotrophic factor in vivo. *Brain Res.* 815 317–325. 10.1016/s0006-8993(98)01100-7 9878807

[B46] Yoshida N. Liberman M. C. (2000). Sound conditioning reduces noise-induced permanent threshold shift in mice. *Hear Res.* 148 213–219. 10978838 10.1016/s0378-5955(00)00161-1

[B47] Zuo H. Cui B. She X. Wu M. (2008). Changes in guinea pig cochlear hair cells after sound conditioning and noise exposure. *J. Occup Health* 50 373–379. 10.1539/joh.L8032 18654041

